# Liquid Crystal Enabled Dynamic Nanodevices

**DOI:** 10.3390/nano8110871

**Published:** 2018-10-23

**Authors:** Zhenhe Ma, Xianghe Meng, Xiaodi Liu, Guangyuan Si, Yan Jun Liu

**Affiliations:** 1 College of Information Science and Engineering, Northeastern University, Shenyang 110004, China; mazhenhe@163.com (Z.M.); siguang@mail.ustc.edu.cn (X.M.); youjing56789@gmail.com (X.L.); 2 Melbourne Centre for Nanofabrication, Victorian Node of the Australian National Fabrication Facility, Clayton, VIC 3168, Australia; 3 Department of Electrical and Electronic Engineering, Southern University of Science and Technology, Shenzhen 518055, China

**Keywords:** liquid crystals, metasurfaces, plasmonics, actively tunable nanodevices

## Abstract

Inspired by the anisotropic molecular shape and tunable alignment of liquid crystals (LCs), investigations on hybrid nanodevices which combine LCs with plasmonic metasurfaces have received great attention recently. Since LCs possess unique electro-optical properties, developing novel dynamic optical components by incorporating nematic LCs with nanostructures offers a variety of practical applications. Owing to the large birefringence of LCs, the optical properties of metamaterials can be electrically or optically modulated over a wide range. In this review article, we show different elegant designs of metasurface based nanodevices integrated into LCs and explore the tuning factors of transmittance/extinction/scattering spectra. Moreover, we review and classify substantial tunable devices enabled by LC-plasmonic interactions. These dynamically tunable optoelectronic nanodevices and components are of extreme importance, since they can enable a significant range of applications, including ultra-fast switching, modulating, sensing, imaging, and waveguiding. By integrating LCs with two dimensional metasurfaces, one can manipulate electromagnetic waves at the nanoscale with dramatically reduced sizes. Owing to their special electro-optical properties, recent efforts have demonstrated that more accurate manipulation of LC-displays can be engineered by precisely controlling the alignment of LCs inside small channels. In particular, device performance can be significantly improved by optimizing geometries and the surrounding environmental parameters.

## 1. Introduction

A surface plasmon resonance (SPR) [1,2,3,4,5,6,7] is formed when the incident electrons resonate with the vibration on the surface of metallic nanostructures, which has caused broad interest regarding novel applications [8,9,10,11,12,13,14,15,16,17,18,19]. Researchers have shown that plasmonic crystals [20,21] are essential for superlenses [22,23], negative refraction applications [24,25], and ultra-large nonlinearity devices [26,27]. Plasmonic metasurface-based devices [28,29,30,31,32,33,34] have demonstrated increasing potential practical applications citing their unique optical characteristics, which can be chosen to be more favorable than natural materials [35,36,37]. Since the experimental demonstration of exotic negative refraction, the concept of metamaterials has been widely favored in the scientific community, especially for enhancing polarization control [38,39,40,41,42], absorption [43,44,45], asymmetric transmission [46,47,48], cloaking [49,50,51], slow light generation [52,53], and novel sources of coherent radiation [54,55]. Similarly, metasurfaces can be easily fabricated using either a top-down or bottom-up approach, and their optical properties can be externally controlled via hybridization with a naturally available functional material, significantly expanding the range of potential practical applications [56,57]. 

So far, several different tuning principles [58,59,60,61,62] based on various types of liquid crystal (LC) molecules, such as chiral [63], nematic [64], and smectic [65] characteristics have been experimentally demonstrated to realize dynamically controllable nanodevices. Since LC-based modulation mechanism can offer an additional advantage because the LC-molecules exhibit a large optical anisotropy [66,67,68,69,70,71,72,73,74,75,76,77,78,79,80,81,82,83], investigations on LCs combined with nanostructures [84,85,86] or two-dimensional materials [87] have recently drawn significant attention and interest. Developing tunable optical metamaterials by incorporating nematic LCs as an electro-optic or nonlinear optical constituent has become a popular research topic [88,89,90,91,92,93]. Given the large birefringence of LCs, the optical properties of these metamaterials can be electrically or optically manipulated through a wide frequency range. Shrekenhamer and coworkers [90] have demonstrated electronically tunable metamaterial absorbers in the terahertz (THz) regime. By incorporating the active LCs into the metamaterial unit cell, the absorption was modified by 30 percent at 2.62 THz, and the resonant absorption could be tuned over 4 percent in bandwidth. A spatial modulator was achieved by Savo et al., using isothiocyanate-based LCs in the THz range [91]. Moreover, by adopting an external voltage, electro-optic switching devices have been achieved via reflection and refraction of LC-cells [92,93]. In addition, LCs are also capable of enhancing the angle reliance of a localized plasmon resonance, enabling ultra-sensitive detection of the resonance shift in the visible and near-infrared regimes [94]. Here, we summarize recent achievements of dynamically tunable nanodevices based on LCs. Given their fantastic optical properties, LCs have found extensive applications, most commonly in display techniques (high display quality, low electromagnetic radiation, large visible area, and low power consumption). On the other hand, metasurfaces offer the advantage of a significant reduction in sizes and physical dimensions and can achieve complex functionalities with simple, elegant designs. Therefore, hybrid optoelectronic devices may be enabled to realize more varied functionalities and a wider range of practical applications by combining LCs and metasurfaces.

## 2. Manipulating LCs and Interaction of LCs with Metasurfaces

### 2.1. Transmission of LCs Covered Metasurfaces

Metasurfaces have become a ubiquitous instrument for low-loss operation of phase, intensity, and polarization of light. Since they can effectively control the flow of light, many applications have been explored from microwave to visible frequencies, including lenses, beam deflectors, and hologram devices. Recent research [95] has presented dynamic manipulation on the optical response of Mie-resonant metasurfaces by controlling the alignment of LCs with an electric field. 

**Figure 1:**
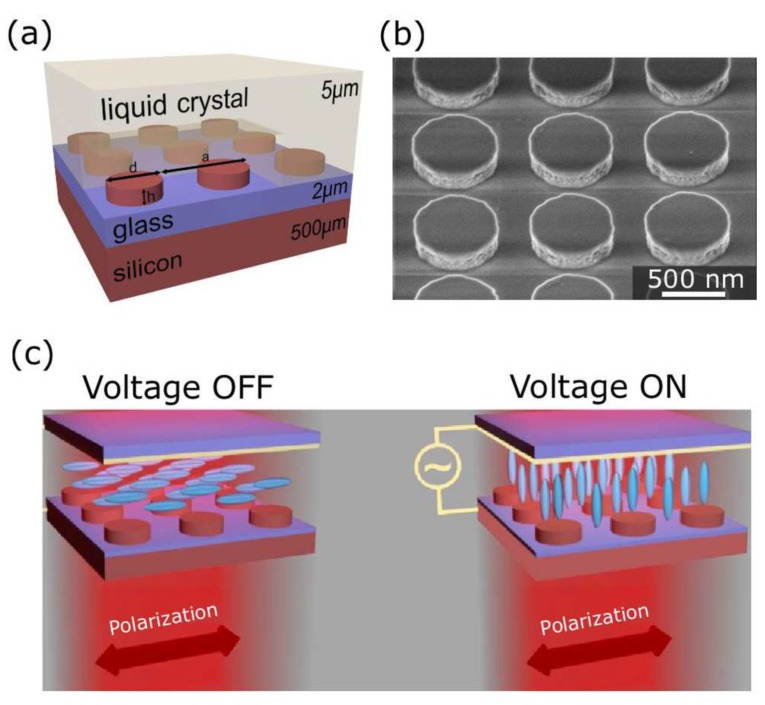
(**a**) Schematic drawing of hybrid structures of nanodisk metasurfaces covered with liquid crystals (LCs). (**b**) Scanning electron microscopy (SEM) image of silicon nanodisks. (**c**) Schematic diagram showing the working mechanism. Reproduced with permission from [95]. Copyright American Institute of Physics, 2017.

As shown in Figure 1a,b, silicon nanodisks can be finely integrated into the LCs. The height and diameter of the nanodisks were 220 nm and 606 nm with a lattice constant of 909 nm. Note that the extraordinary and ordinary indices of nematic cells used were 1.7 and 1.51, respectively. In addition, the internal thickness of the LC-layer was fixed at 5 μm using a suitable spacer material, whilst the substrate was used as an electrode. The preferred orientation of the LC cells was induced by brushed Nylon-6. For further verification, when the voltage was “OFF”, the LC alignment was parallel with the metasurface. To achieve the switching effect, the LC molecules can be redirected perpendicular to the metasurface when the voltage was turned “ON”.

**Figure 2:**
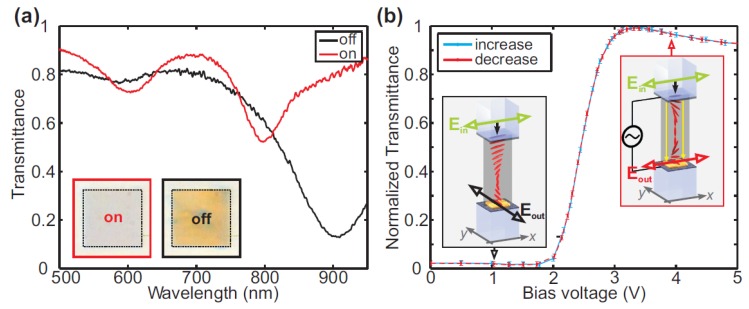
(**a**) Measured transmission of LC-covered metasurfaces when the external electric field is on and off. The insets demonstrate captured charge-coupled device (CCD) images showing the color difference from yellow to almost transparent. (**b**) Normalized transmittance as a function of bias voltage. The threshold behavior can be clearly observed for the switching process. Insets show schematic of LC-cells alignment without (left) and with (right) electric field. Reproduced with permission from [99]. Copyright Optical Society of America, 2013.

Thus far, most investigations have been focused on the active control of metasurfaces immersed in nematic LCs. It has been found that one can manipulate the alignment of LC-cells by applying an external electric field, which results in a significant exponential variation in a series of plasmonic devices [96,97,98]. Decker and coworkers have shown active control on transmission spectrum of LC-coated metasurfaces [99]. With an x-polarized incident beam, the transmittance spectrum was measured. There is only electric resonance at the wavelength of 900 nm without a bias voltage (*V* = 0 V), as shown in Figure 2a. After applying a 6 V external voltage, the device shows two magnetic resonances at wavelengths of 600 nm and 800 nm, respectively. Furthermore, the color change from yellow to almost transparent between “OFF” and “ON” states can be observed, as shown in the inset of Figure 2a. Due to the reorientation of LC-cells driven by the external electric field, the incident light had to rotate 90° to form a helical distribution when passing through the hybrid device. When the bias voltage was switched on, the LC molecules between the two electrodes were redirected (parallel with the electric field), disrupting the helical distribution. Figure 2b plots the threshold characteristic of the working principle of this active device. One can see that the normalized transmittance increases dramatically between 2 and 3 V, producing the switching effect. Once the voltage reaches 5 V, the light transmittance is saturated and the spectrum remains unchanged.

### 2.2. Transmission of PDLCs Controlled by Surface Acoustic Waves

**Figure 3:**
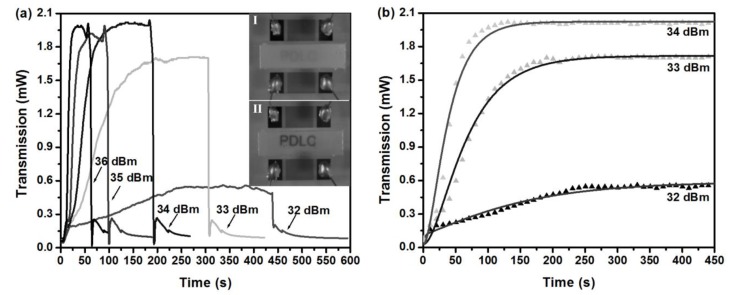
(**a**) Transmission as a function of time showing the switching process with varying power of surface acoustic waves (SAWs). The insets illustrate that the polymer-dispersed liquid crystal (PDLC) film is opaque without SAW and becomes transparent with SAW. (**b**) The enlarged view with time from 0 S to 450 S showing the switch-on process with theoretical fitting. Reproduced with permission from [86]. Copyright John Wiley & Sons, 2011.

For the past few years, polymer-dispersed liquid crystals (PDLCs) have found wide applications in a variety of fields. The orientation of LC-cells can be realigned by applying an external electric field, and therefore, the refractive index difference can be precisely modulated between cells and the polymer matrix, which means PDLCs can be shifted between transparent and opaque phases. Using surface acoustic waves (SAWs), Liu and coworkers [86] have demonstrated that one can match the refractive index between LC-cells and the polymer matrix, and therefore, achieve a switching effect. Accurate active control can be realized to drive the LC-cells to a specific orientation. Figure 3a plots the transmission of the PDLC film as a function of time with varying acoustic intensities, and the switching effect is observed clearly from the inset. Unperturbed, the PDLC film was opaque. After applying a SAW, the PDLC film became transparent and letters of “PDLC” were revealed. Note that a low SAW intensity may result in a long switch-on time with a correspondingly small optical contrast ratio. Alternatively, the switch-off time can be significantly increased by reducing the size of the droplets. For example, a PDLC-film with a uniform micron level droplet (1–3 μm) may lead to a switch-off time in the milliseconds scale. Furthermore, the driving power can be effectively decreased by adding a surfactant to PDLCs, which can act as a lubricating reagent. From Figure 3b, a clear switching effect is shown where a double exponential function is employed to fit the experimental curves, as in Reference [86]:

(1) $$I(t)=I_{\min}+I_{0}\sin^{2}\left\{\frac{1}{2}\delta_{1}\left[1-\exp\left(-t/\tau_{1}\right)\right]+\frac{1}{2}\delta_{2}\left[1-\exp\left(-t/\tau_{2}\right)\right]\right\}\;$$

where *δ*_i_ and *τ*_i_ (*i* = 1, 2) denote the fitting parameters. It is clear that Figure 3b shows the dynamic switching behavior which can be well-fitted by the double exponential function.

### 2.3. Transmission of Coaxial Color Filters Covered with Photoresponsive LCs

**Figure 4:**
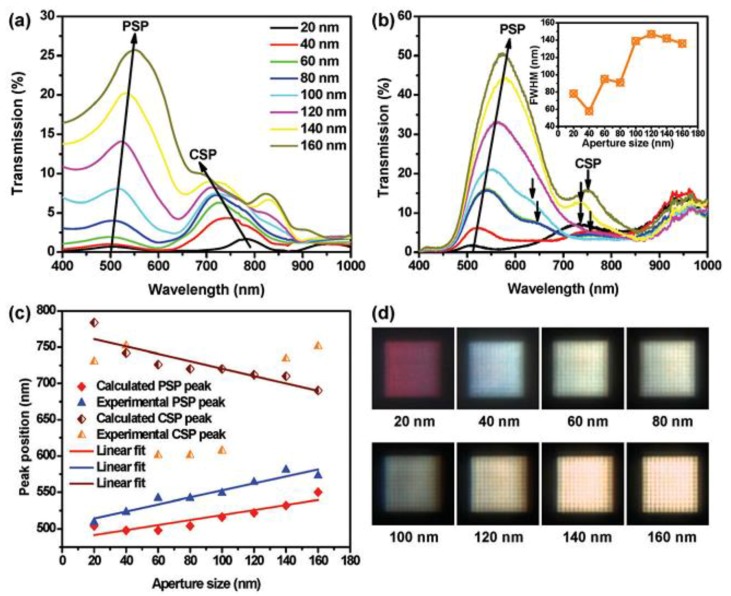
(**a**) Simulated and (**b**) experimental results of transmission for annular aperture arrays (AAAs) designed in a gold film. Aperture size increases from 20 nm to 160 nm in step of 20 nm. (**c**) Peak position as function of aperture size showing both cylindrical surface plasmons (CSPs) and planar surface plasmons (PSPs) peaks with linear fit. (**d**) CCD images captured by using an optical microscope. Reproduced with permission from [80]. Copyright John Wiley & Sons, 2012.

Recently, plasmonic color filters based on annular aperture arrays (AAAs) were experimentally demonstrated by manipulating their geometric parameters [100,101]. However, the transmitted intensity was low because of large propagation losses, resulting in weak coupling between neighboring annular apertures. Most structural color filters are passive, which means the output is constant once the design is defined. Therefore, developing active color filtering devices with small pixels and high efficiency is crucial for new display techniques. By combining plasmonic nanopixels with photoresponsive LCs, all-optical color filters have been demonstrated with enhanced transmission [80]. Plasmon resonance peaks can be tuned across the whole visible band, allowing individual color outputs to be filtered out from a white light source. As shown in Figure 4a, two different resonance peaks are contained in the wavelength range of 500–800 nm, which are known as planar surface plasmons (PSPs) and cylindrical surface plasmons (CSPs), respectively. For small aperture widths (20, 40, 60, and 80 nm), one can see that CSPs are dominant. When the aperture size is 100 nm, CSPs and PSPs show similar intensity (around 7.5%). With further increased aperture widths, PSPs contribute more and grow faster than CSPs peaks. Calculations in Figure 4a, also indicate the trend of the redshift of PSPs with increasing aperture widths. Experimentally measured spectra in Figure 4b, show similar intensity change and resonance positions for PSPs peaks. However, CSPs show random positions due to fabrication imperfections. The transmission intensity of the short wavelength range is very different. Moreover, it can be seen from Figure 4c that the intensity of CSP is higher than PSP for smaller aperture sizes. Note that PSP-induced propagation gradually plays a leading role with increasing aperture sizes, and their full width at half maximum (FWHM) grows and finally reaches saturation. Figure 4c shows the peak position of the transmittance as a function of aperture size. In addition, the transmission peak induced by PSPs linearly redshifts. The transmission peak induced by CSPs linearly blueshifts with larger aperture sizes. Figure 4d illustrates CCD captured images and color changes from blue to red as aperture size grows, except for the 20 nm apertures, which show carmine because they are dominated by CSPs. Relatively high color crosstalk in measured results is because the peaks induced by PSPs and CSPs do not completely overlap with each other. Nevertheless, device performance can be further improved by carefully designing structural parameters.

### 2.4. Transmission of LC Coated High Aspect Ratio Nanorods

**Figure 5:**
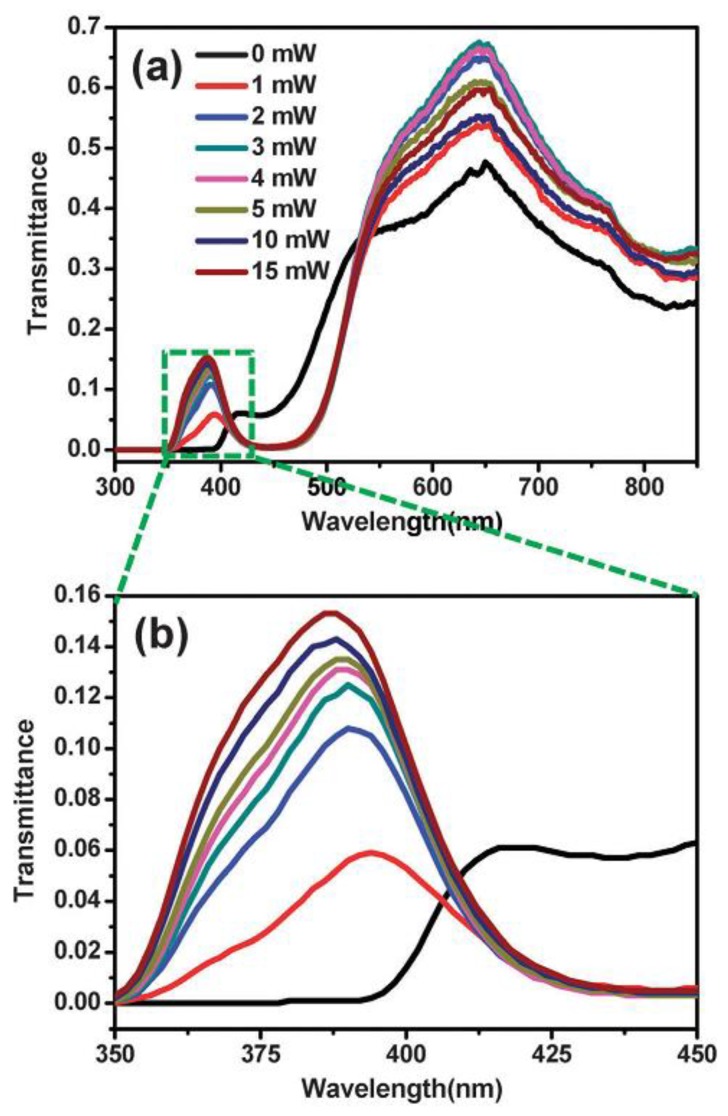
(**a**) Measured transmittance of silver nanorods covered by LCs as a function of wavelength for varying pumping power at 20° incidence angle and (**b**) enlarged view of 350 nm to 450 nm wavelengths showing more details of resonances located at 380–390 nm. Reproduced with permission from [102]. Copyright Royal Society of Chemistry, 2015.

One main obstacle to achieve high-efficiency hybrid devices using LCs and functional nanodevices is the question of how to combine them. Geometry may affect the orientation of LC cells significantly. High aspect ratio nanorods can align well with LC molecules. Using silver nanorods with large heights, it has been shown that it is possible to achieve homeotropic status [102]. In general, the transmission changes are based primarily on the excitation of the plasmonic nanorods. The transmission characteristics of the incident angle on the hybrid system have been thoroughly investigated. Experimental results demonstrate clear power dependence via transmission measurements. Figure 5 shows the effect of pumping power on transmittance at a 20° angle of incidence. As plotted in Figure 5a, one can see that the transmitted intensity is enhanced dramatically with growing pumping power until it saturates at around 10 mW. From the wavelength range of 400–500 nm, spectra show negligible changes with pumping light due to low transmitted intensities. It should be noted that more energy can pass through the hybrid system at longer wavelengths, resulting in a peak at ~650 nm. Figure 5b plots the magnified view of Figure 5a in the range of 350–450 nm, and another transmission peak between 380 nm and 390 nm can be seen as the pumping power is increased. Without pumping, the original resonance locates at around 420 nm and then shifts to 390 nm after switching on the pumping light. With further increase to pumping power, transmittance grows significantly from 6% (1 mW) to 15% (15 mW) with slight blueshift of resonance wavelength. 

### 2.5. Reflective Metasurface Lenses

**Figure 6:**
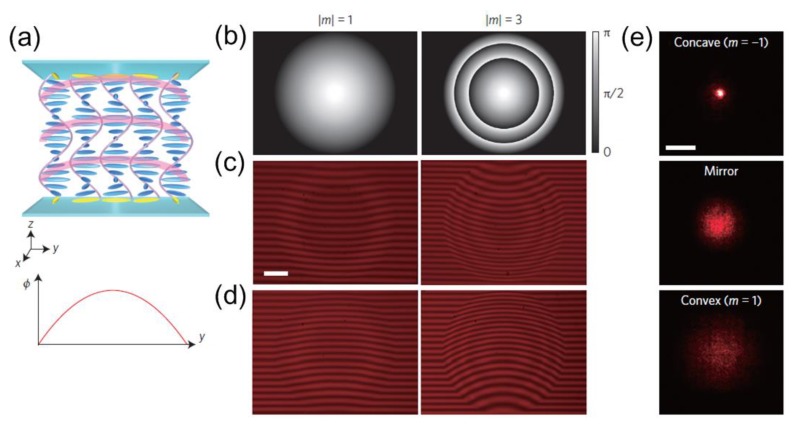
(**a**) Sketch of the working mechanism of metasurface based reflective lenses combined with patterned cholesteric liquid crystals (ChLCs) and the corresponding phase profile. (**b**–**d**) Device performance for different phases of π and 3π. Scale bar denotes 200 µm. (**e**) Recorded laser spot reflected from different interfaces compared with a mirror. Concave and convex profiles have been recorded for *m* = −1 and *m* = 1, respectively. Scale bar denotes 2 mm. Reproduced with permission from [108]. Copyright Springer Nature, 2016.

Reflective metasurfaces [103,104,105,106,107] have recently attracted great interest due to their ability of manipulating electromagnetic waves at the nanoscale. Since these nanodevices demand sub-wavelength characteristics, components operating in visible frequencies are normally difficult to fabricate. Kobashi et al., [108] have shown that chiral LCs with a self-organized helical structure can lead to non-specular reflection at optical frequencies. Figure 6 shows the working principle of reflective lenses with patterned cholesteric liquid crystals (ChLCs). Figure 6a plots a reflective Fresnel lens with a parabolic phase profile wrapped to π. A functional area with circular shape was fabricated with 1 mm diameter for different phases of π and 3π, as demonstrated in Figure 6b–d. Light reflection can be produced at two different interfaces. Thus, the phase profile can be reversed in accordance with different observation direction, enabling converging or diverging effect according to the angle of incidence. As shown in Figure 6e, the laser spot shows different profiles at varying reflected surfaces compared with a mirror. Concave and convex profiles have been recorded for *m* = −1 and *m* = 1, respectively. Note that the reflective lens using ChLCs is completely reconfigurable because the convergence or divergence status can be switched freely by simply flipping the sample. The focal length is determined by Fresnel’s approximation, which is related to aperture radius and phase difference. In this case, calculated focal length is 20.3 cm, which agrees well with the experimental results. This means extensive useful planar reflective optical components are enabled by using similar optoelectronic designs with ChLCs.

## 3. LC-Based Plasmonic Tunable Applications

In this Section, we review recent interesting tunable devices enabled by LC-plasmonic interactions, which have demonstrated practical applications ranging from resonance tuning to dynamic color switching.

### 3.1. Localized Surface Plasmon Resonance (LSPR) Tuning with Holographic Polymer-Dispersed Liquid Crystals (HPDLCs)

**Figure 7:**
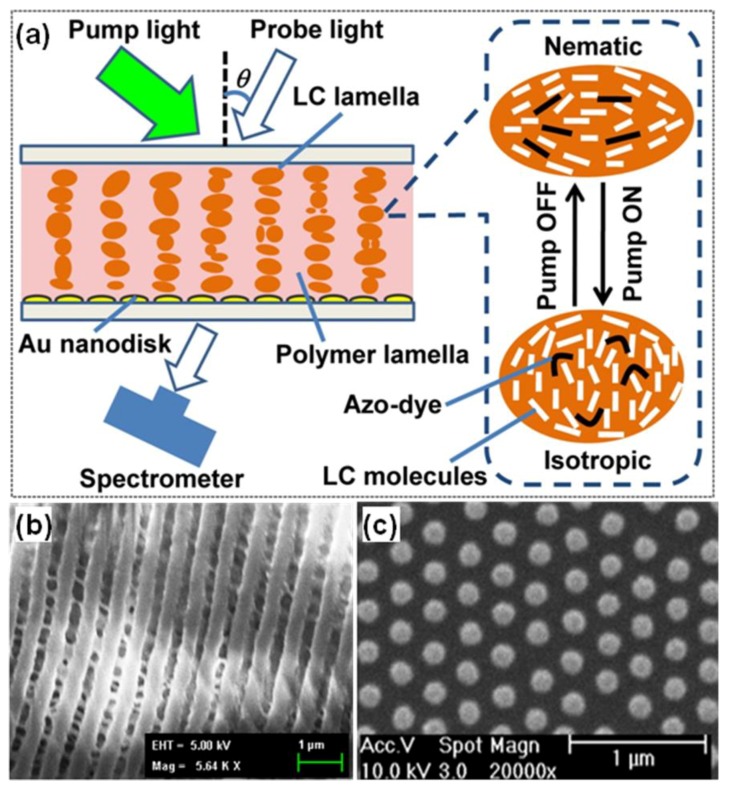
(**a**) Working principle of holographic polymer-dispersed liquid crystals (HPDLCs) transmission grating covered gold nanodisks. Modulation of resonance is realized by using a pumping light. The right part shows magnified view which demonstrates HPDLCs can be shifted between nematic and isotropic with pumping light off and on. (**b**) SEM topography of the HPDLCs transmission grating and (**c**) top-view SEM image of gold nanodisks fabricated by nanolithography followed by reactive ion etching with ~150 nm diameter and ~320 nm periodicity. Reproduced with permission from [109]. Copyright American Chemical Society, 2011.

Until now, investigations on manipulating plasmon resonance have been performed and most of the achievements take advantage of tuning structural parameters and dielectric environment, with different complex designs. One main problem is that these devices normally show poor reproducibility and very limited tunable range. To further improve device performance and achieve highly reproducible results, azo-dye based holographic polymer-dispersed liquid crystals (HPDLCs) can be employed [109]. The hybrid system combines gold nanodisks with HPDLCs, which can be switched between nematic and isotropic with pumping light off and on. Figure 7a shows the principle diagram of the experimental device, which contains gold nanodisks covered with a HPDLC-layer where its orientation is controlled by an external optical field. Under light irradiation, the grating structure may suffer from thermal expansion due to localized heating of the functional area. The LC-droplets can be further squeezed, producing a shape change from oval to spherical. Scanning electron microscopy (SEM) topography of the HPDLCs transmission grating is shown in Figure 7b, with around 600 nm pitch. The nanodisk array was fabricated on a glass substrate using nanolithography, followed by reactive ion etching with ~150 nm diameter and ~320 nm periodicity.

**Figure 8:**
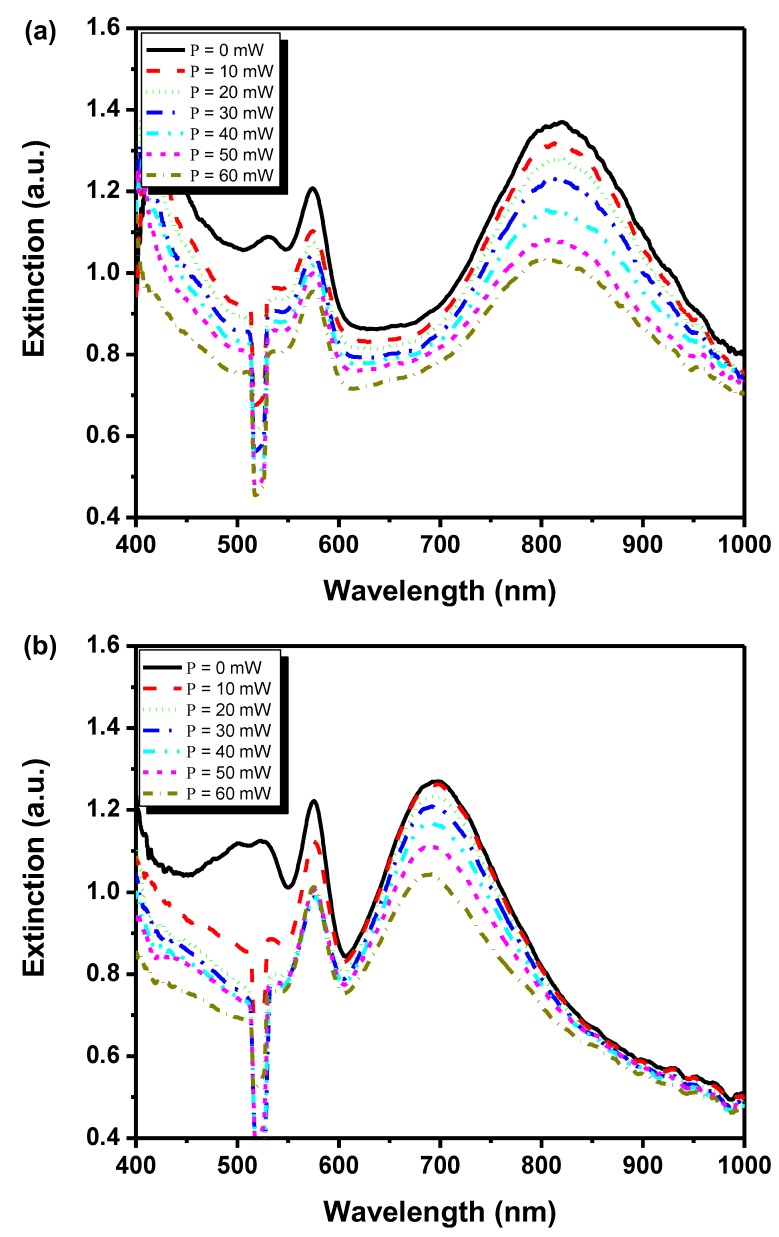
Measured extinction spectra as a function of wavelength with varying pumping power for (**a**) coupled system (nanodisks with HPDLCs transmission gratings), and (**b**) uncoupled nanodisks (separated from HPDLCs transmission gratings). Reproduced with permission from [109]. Copyright American Chemical Society, 2011.

As for the device shown in Figure 7, the measured extinction spectra at different pumping intensities are shown in Figure 8a for an incident probe angle of 42 degrees. Note that the extinction can be enhanced by decreasing pumping power with a slight redshift of resonance from around 800 nm (60 mW) to 830 nm (0 mW). However, the peak located at 580 nm remains unchanged with varying pumping power. For comparison, a control experiment was carried out which performed extinction measurement of nanodisks separated from the HPDLCs transmission gratings, as plotted in Figure 8b. It can be observed that the extinction peak significantly blueshifts to about 700 nm, compared with the hybrid system of nanodisks with the HPDLCs transmission gratings. The resonance peaks are also narrower. A lower intensity of resonance peaks compared with the coupled hybrid system is observed. However, the trend of intensity changes for peaks at longer wavelengths (~700 nm), shows similar behavior of increment extinction with smaller pumping power. For the resonance located at shorter wavelengths (~580 nm), extinction reaches saturation after the pumping power is larger than 20 mW. The coupled hybrid system also shows a periodic modulation in the absorption spectrum, forming an absorption grating, and therefore, resulting in diffraction of more light near the localized surface plasmon resonance (LSPR) peaks.

**Figure 9:**
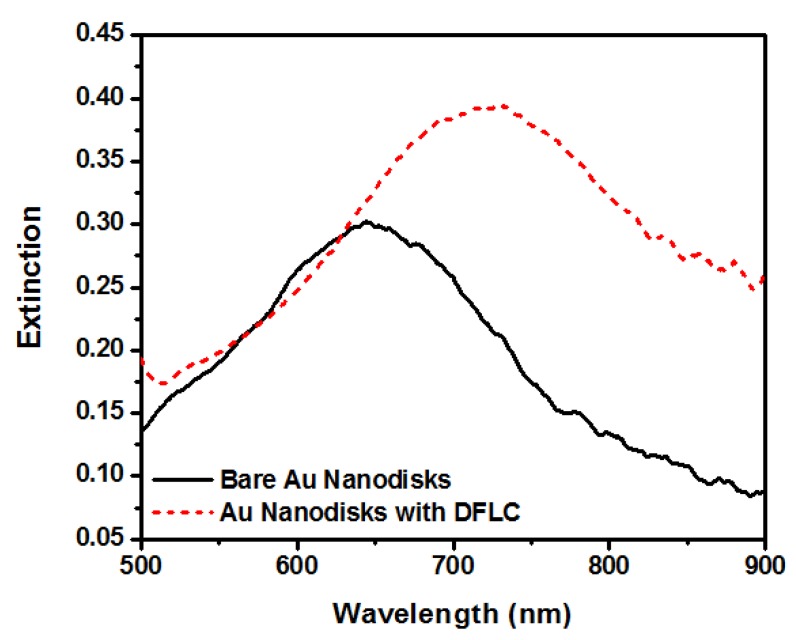
Comparison of measured extinction of nanodisks at normal incidence with and without dual-frequency liquid crystals (DFLCs). Reproduced with permission from [110]. Copyright American Institute of Physics, 2010.

To further investigate the underlying physics, nanodisks covered with dual-frequency liquid crystals (DFLCs) have been measured compared with bare gold disks, as shown in Figure 9. Note that this measurement was performed under normal incidence. The solid black and dashed red curves show different intensities and peak positions. Increased intensity with redshifted (~80 nm) resonance after combining gold nanodisks with DFLCs can be seen. 

### 3.2. Temperature-Dependent Tuning with Thermotropic LCs

**Figure 10:**
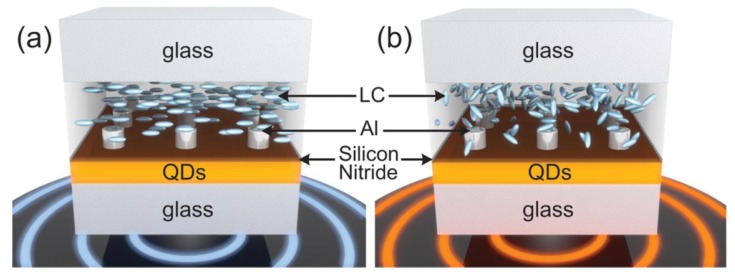
Schematic diagram of the temperature-dependent hybrid system using thermotropic LCs. (**a**) LC molecules are ordered at room temperature. (**b**) LC molecules are disordered at higher temperatures (>58 °C). Reproduced with permission from [111]. Copyright American Chemical Society, 2014.

Using thermotropic LCs, Abass and coworkers have shown an active tuning function which is temperature dependent [111]. Figure 10 shows the sketch of the experimental setup at different temperatures. It represents the diverse states of LCs. First, quantum dots (QDs) were synthesized by means of chemical processes of a consecutive ion layer absorption and reaction procedure. Beginning with an average QDs diameter of 6.5 nm, a 120 nm thick QD layer was obtained after coating on a glass substrate. A 15 nm thick silicon nitride layer was subsequently deposited on the QD layer for protecting the waveguide and smoothing the surface. Then aluminum nanoantennas were fabricated using nanoimprint lithography. Finally, thermotropic LCs were coated and a nylon alignment material was introduced to ensure that the orientation was in the direction of its lattice vectors.

**Figure 11:**
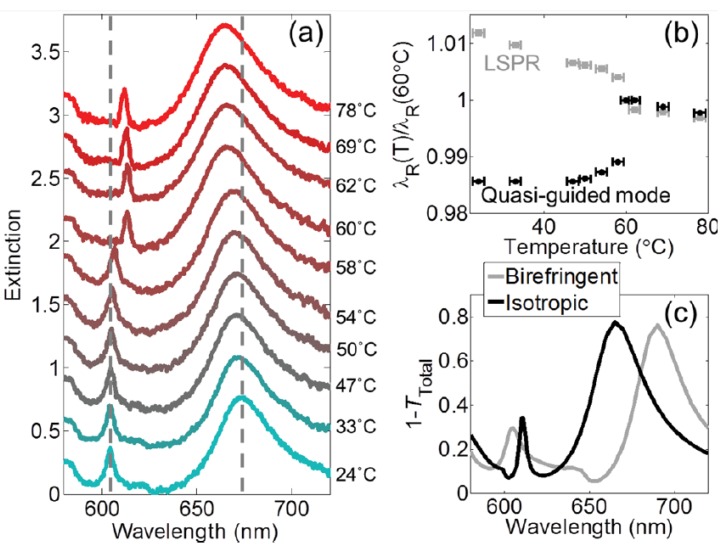
(**a**) Measured extinction as a function of wavelength at different temperatures. (**b**) Temperature-dependent normalization of resonance wavelength to its corresponding wavelength at 60 °C for localized surface plasmon resonance (LSPR) and quasi-guided modes. (**c**) The simulation spectra of two states, where the temperatures of the LCs are set to 24 °C and 60 °C, respectively. Reproduced with permission from [111]. Copyright American Chemical Society, 2014.

Figure 11a shows the measured extinction spectra at various temperatures of the device shown in Figure 10. A halogen lamp was used as a broadband light source to illuminate the sample, thereafter the zeroth order transmittance *T*_0_ was measured by a fiber-coupled spectrometer. There are two peaks observed in Figure 11a, which are in accordance with the hybrid plasmonic-photonic resonances. The wide resonance at long wavelengths results from the LSPRs. On the other hand, the narrow resonance at short wavelengths derives from the quasi-guided modes. Figure 11b illustrates the temperature-dependent normalized values of two modes. Interestingly, it can be seen the tendencies vary distinctly from the LSPRs to quasi-guided modes. As the transition happens, the anisotropic refractive index takes the place of the birefringent index. Figure 11c shows the 1−*T*_total_ spectra in the state of ordered and disordered LCs, respectively. The peak resonance wavelengths shown in the simulated curves are found to be in good agreement with the experimental results.

### 3.3. Active Tuning of the Fano Resonance

**Figure 12:**
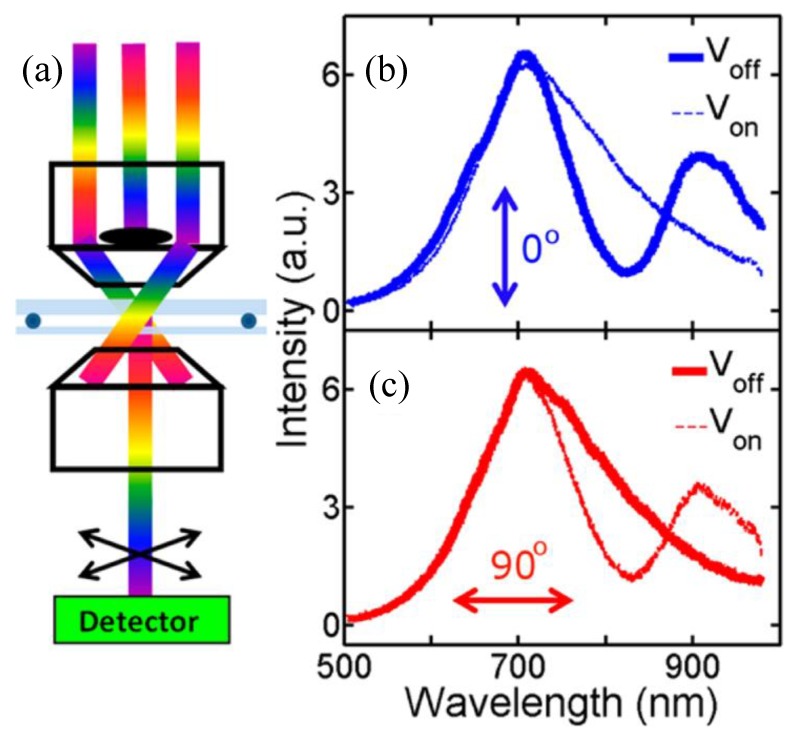
Dynamic manipulation of the Fano resonance using LCs. (**a**) Schematic diagram of the experimental setup of dark field microspectroscopy. (**b**,**c**) Measured scattering spectra as a function of wavelength for an individual octamer nanostructure at different polarization of (**b**) 0° and (**c**) 90°. Reproduced with permission from [117]. Copyright American Chemical Society, 2012.

The collective oscillation of electrons at the interface of metal and dielectric is called surface plasmon. When neighboring nanoparticles are strongly coupled with each other, they can exhibit unique line shapes and interference, such as the famed Fano resonance. The Fano resonance is highly dependent on environmental parameters, which can be used to significantly increase the figure of merit for LSPR sensing [112,113,114,115,116,117,118]. Figure 12 demonstrates the working principle of a dynamic device which can control the Fano resonance using LCs [117]. In this experiment, an unpolarized white light source passed through a dark-field condenser and then focused on the sample surface. An oil-immersion objective lens was used to collect the light scattered by the nanostructures. Scattering spectra in Figure 12b were obtained at 0° polarization direction. The solid blue curve represents a Fano-like spectrum without external electric field (*V*_off_). After applying a voltage to the LCs device, the Fano resonance is switched off. Similar switching behavior can be obtained at 90° polarization direction as well, as shown in Figure 12c. However, one can see that there is no Fano resonance at the *V*_off_ state. After switching on the voltage, a Fano-shaped profile is revealed, as shown by the red dashed line.

### 3.4. Dynamic Color Tuning

**Figure 13:**
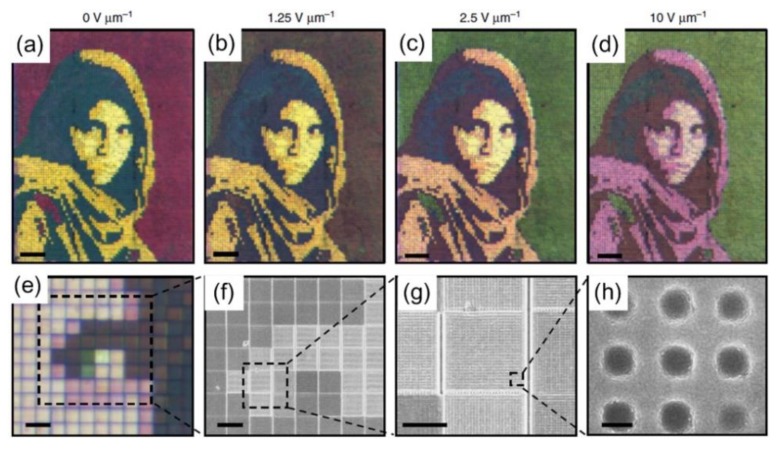
Dynamic color tuning using LCs. (**a**–**d**) Microscope images of captured portrait at varying applied voltages. (**e**) Magnified image at 10 Vμm^−1^ showing more details. (**f**–**h**) SEM images of fabricated structural pixels. Scale bars, (**e**) 20 μm, (**f**) 10 μm, (**g**) 5 μm, (**h**) 150 nm. Reproduced with permission from [119]. Copyright Macmillan Publishers Limited, 2015.

Compared to traditional display technologies, there are many benefits of structural color filtering devices, including higher resolution and smaller pixels. However, their potential applications are limited because their optical characteristics remain static. By combining elaborately designed metasurfaces with highly birefringent LCs, an active reflection-type color filter has been demonstrated [119], which is not dependent on polarization. Combining the nanoimprinted structures with different geometric parameters with LCs, one can realize dynamic color tuning over the entire visible regime. As shown in Figure 13, the combination of LCs with metasurfaces can produce different color outputs [119]. Moreover, 10 µm × 10 µm pixels in the accompanying SEM images can be considered as two-dimensional gratings with different structural parameters. Such LC-plasmonic hybrid systems can be simply integrated with other display techniques. To prove this capability, the traditional transparent LCs display panel is applied, and the hybrid system can be integrated with a commercially-available thin-film-transistor (TFT) array. The aluminum metasurface was fabricated on the TFT glass plate with an 8.5 µm spacer layer, and then filled with high birefringence LCs [120]. 

**Figure 14:**
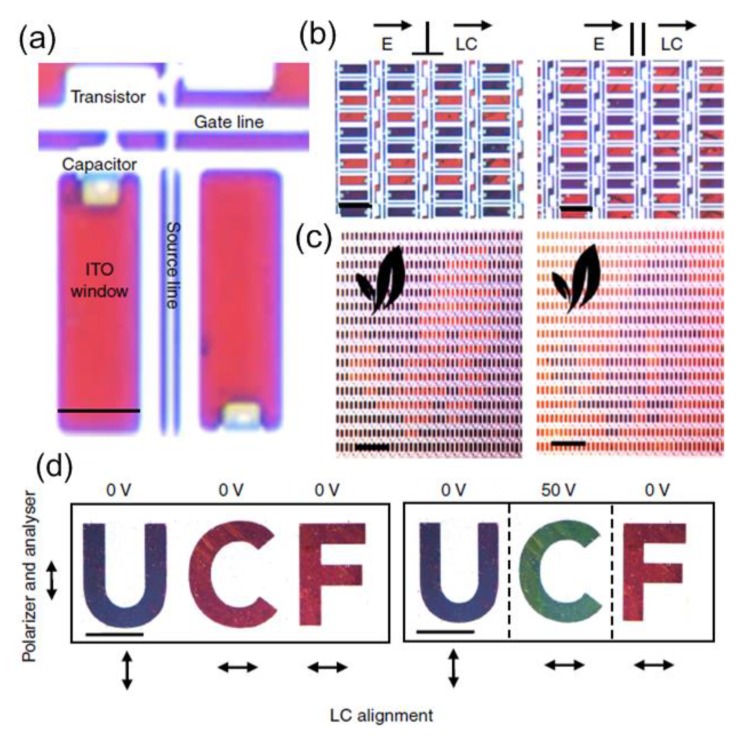
(**a**) Microscopic image of metasurfaces integrated with thin-film-transistor (TFT), and (**b**) shows that each row is opened every third and fourth rows in the case of parallel and vertical incident light for the top LCs orientation layer. (**c**) Arbitrary image displayed using a device photographed with a 4× objective. (**d**) Passive addressing devices with defined letters of “UCF” as a function of different parameters. Reproduced with permission from [120]. Copyright Macmillan Publishers Limited, 2017.

Figure 14a exhibits the device, and the electrical components of TFT are seen. Light can pass through the polarizer, LCs, and indium tin oxide (ITO) window to reflect the surface. Figure 14b shows color change with an applied voltage. Figure 14c illustrates an arbitrary shaped object captured with a 4× objective lens. The nanostructured surface is macroscopically patterned using UV lithography, followed by aluminum cladding. For this surface, the top ITO glass was patterned to control each letter of “UCF” individually. Moreover, a UV photo alignment was used to show how the *V*_off_ colors could be manipulated. Under a linearly polarized source, the azobenzene material can make the LCs uniform and perpendicular to the polarization direction.

### 3.5. Narrowband Reflection Filter Controlled by LCs

**Figure 15:**
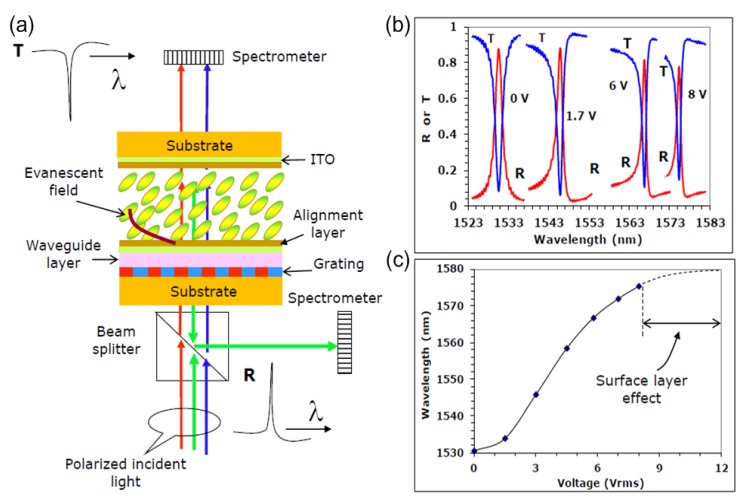
(**a**) Sketch of the hybrid device of reflection filter which can be controlled by nematic LCs. (**b**) Transmitted and reflected spectra at varying voltages. (**c**) Tunability curve of resonance wavelength as a function of voltage. S-shaped profiles can be observed, which means the nanoscale coherence region cannot switch with a fast speed. Reproduced with permission from [121]. Copyright Society of Photographic Instrumentation Engineers (SPIE) and Chinese Optics Letters (COL), 2012.

Nowadays, with the development of powerful computer simulations, the design of LC-based devices can be applied even at the molecular level. More novel tunable devices can be created by combining optical components with LCs. Figure 15 shows the working principle of a narrowband resonant reflection filter which can be controlled by nematic LCs [121,122]. In this case, the coherence region was mainly probed by the evanescent field when LC cells were placed on a waveguiding layer. Transmitted and reflected spectra at varying bias voltages are plotted in Figure 15b. Owing to the strong anchoring, the resonance wavelength as a function of voltage shows S-shaped profiles (see Figure 15c for more details), which means the nanoscale coherence region cannot switch with a very fast speed.

## 4. Conclusions and Outlooks

To conclude, we have reviewed the recent development of tuneable devices based on LC-plasmonic metasurfaces and their potential applications. Citing recent development of metasurfaces, as well as the exploration of new LCs functions, actively tuneable metamaterial-based devices and more practical achievements have been developed which can pave the way for new plasmonic display technologies. Nevertheless, we still need to ameliorate the sometimes less-than-ideal optical performance of these hybrid devices to achieve more varied and useful applications via consistent and reliable components. Additionally, high speed dynamic devices are of great importance for future development of multiple-functional optoelectronic nanodevices. These active nanodevices may enable more innovations due to their capability of manipulating electromagnetic waves freely at the nanoscale.
